# Influence of comorbidities in idiopathic normal pressure hydrocephalus — research and clinical care. A report of the ISHCSF task force on comorbidities in INPH

**DOI:** 10.1186/2045-8118-10-22

**Published:** 2013-06-10

**Authors:** Jan Malm, Neill R Graff-Radford, Masatsune Ishikawa, Bo Kristensen, Ville Leinonen, Etsuro Mori, Brian K Owler, Mats Tullberg, Michael A Williams, Norman R Relkin

**Affiliations:** 1 Department of Clinical Neuroscience, Umeå University, Umeå, 901 85, Sweden; 2 Department of Neurology, Mayo Clinic Jacksonville, Jacksonville, Florida, USA; 3 Normal Pressure Hydrocephalus Center, Otowa Hospital, Kyoto, Japan; 4 Department of Neurology, Aalborg University, Aalborg, Denmark; 5 Department of Neurosurgery, KUH NeuroCenter, Kuopio University Hospital and Institute of Clinical Medicine, Kuopio, Finland; 6 Department of Neurosurgery, University of Eastern Finland, Kuopio, Finland; 7 Department of Behavioral Neurology and Cognitive Neuroscience, Tohoku University Graduate School of Medicine, Sendai, Japan; 8 Department of Surgery, University of Sydney, Sydney, Australia; 9 Institute of Neuroscience and Physiology, The Sahlgrenska Academy, University of Gothenburg, Gothenburg, Sweden; 10 The Sandra and Malcolm Berman Brain & Spine Institute of Sinai Hospital, Baltimore, USA; 11 Department of Neurology and Neuroscience, Weill Cornell Medical College, New York, USA

**Keywords:** Hydrocephalus, Normal pressure, Comorbidity, Review, Guidelines, Task force

## Abstract

Idiopathic normal pressure hydrocephalus (INPH) is a syndrome of ventriculomegaly, gait impairment, cognitive decline and incontinence that occurs in an elderly population prone to many types of comorbidities. Identification of the comorbidities is thus an important part of the clinical management of INPH patients. In 2011, a task force was appointed by the International Society for Hydrocephalus and Cerebrospinal Fluid Disorders (ISHCSF) with the objective to compile an evidence-based expert analysis of what we know and what we need to know regarding comorbidities in INPH. This article is the final report of the task force. The expert panel conducted a comprehensive review of the literature. After weighing the evidence, the various proposals were discussed and the final document was approved by all the task force members and represents a consensus of expert opinions. Recommendations regarding the following topics are given: I. Musculoskeletal conditions; II. Urinary problems; III. Vascular disease including risk factors, Binswanger disease, and white matter hyperintensities; IV. Mild cognitive impairment and Alzheimer disease including biopsies; V. Other dementias (frontotemporal dementia, Lewy body, Parkinson); VI. Psychiatric and behavioral disorders; VII. Brain imaging; VIII. How to investigate and quantify. The task force concluded that comorbidity can be an important predictor of prognosis and post-operative outcome in INPH. Reported differences in outcomes among various INPH cohorts may be partly explained by variation in the rate and types of comorbidities at different hydrocephalus centers. Identification of comorbidities should thus be a central part of the clinical management of INPH where a detailed history, physical examination, and targeted investigations are the basis for diagnosis and grading. Future INPH research should focus on the contribution of comorbidity to overall morbidity, mortality and long-term outcomes.

## Review

### Introduction

#### Background and objective

The International Society for Hydrocephalus and Cerebrospinal Fluid Disorders (ISHCSF) has identified six fields of interest for investigation by individual task forces. The task forces have been charged with providing evidence-based expert analysis of what we know now, and what we need to know in the future, to improve the care of INPH patients and move the field of adult hydrocephalus research forward. This article discusses comorbidities in Idiopathic Normal Pressure Hydrocephalus (INPH). For the purposes of this review, comorbidity was defined as a “medical condition existing simultaneously but independent of INPH” or “a medical condition in a patient that causes, is caused by, or is otherwise related to INPH” [1].

INPH is a complex syndrome of ventriculomegaly, cognitive decline, gait impairment and incontinence that occurs in an elderly population prone to many types of comorbidities. Identification of comorbidities is an important part of the clinical management of INPH patients. For instance, comorbidities such as uncontrolled hypertension and cardiac disease need to be addressed before cerebrospinal fluid (CSF) shunt surgery is performed, as part of the medical clearance for general anesthesia. The presence of comorbidities can be a major prognostic factor in the outcome of INPH treatment. For example, shunt surgery in an INPH patient with multiple strokes or co-existing Alzheimer disease is less likely to yield a fully favorable long-term outcome than in persons with INPH who lack these comorbidities. In an elderly population, INPH may be one of many co-existing chronic comorbid conditions, the management of which can contribute to the patient’s overall health status. Identifying and treating comorbid conditions in addition to INPH serves the goal of maximizing overall health status, an important goal in the care of the elderly.

Comorbidity is also important in hydrocephalus research. In order to compare studies and different samples of patients, the degree of comorbidity has to be quantified. Large INPH studies often focus on outcome of CSF shunt surgery [2-6] or report the usefulness of various kinds of predictive tests for INPH such as CSF infusion tests [2], external lumbar drainage [3,5], intracranial pressure monitoring [4], or MRI [6]. However, none of these studies have evaluated the contribution of comorbidity to the reported results. Future INPH research should investigate the contribution of comorbidity to overall morbidity, mortality and long-term outcome in INPH.

The list of differential diagnoses in INPH is long and complicated because of the comorbidities. This review tries to explain how to recognize if INPH patients have comorbid factors and should help in the management of these persons.

#### Search strategy and evidence review

The task force panel conducted an evidence-based expert review of the literature. PubMed was searched for peer-reviewed papers and reviews published in English and including human subjects. The key search term “hydrocephalus” was combined with the following words: comorbidity, atherosclerosis, stroke, transient ischemic attack, musculoskeletal, hip, knee, myelopathy, spinal stenosis, urinary, incontinence, risk factors (including naming of the most important vascular risk factors), Binswanger disease, white matter, leukoaraiosis, MRI, Alzheimer’s disease, biopsy, biomarker (and naming of the most important CSF biomarkers), dementia, psychiatry, depression, psychosis and atrophy.

The chairman of the Comorbidity Task Force (JM) was appointed by the ISHCSF and selected a group of physicians and researchers with knowledge and experience in this area. The task force was composed of neurosurgeons and neurologists with international representation. Each member was given responsibility for performing a thorough literature search on identified subtopics and drafting the relevant section of the consensus document. After weighing the evidence, the various proposals were discussed by all members of the task force by mail and in web-based teleconferences. The final draft was reviewed by external reviewers from this journal and approved by all the task force members. The document thus represents a consensus of expert opinions. The publication was approved by the ISHCSF.

As there are no randomized clinical studies or meta-analyses about comorbidity in INPH, an evidence-based survey of current practice was the basis for the recommendations given. Thus, the primary sources for the recommendations are the expert consensus opinion, clinical experience, case studies, or standard of care.

For each recommendation, the size and strength of treatment effect was categorized as level I, IIa, IIb or III. If level I, the treatment or procedure should be performed. For level IIa the treatment or procedure is reasonable, for IIb, treatment may be considered and for level III, the treatment or procedure would have no effect or could be dangerous for the patient. Those numbers do not correspond to a level of evidence, but to a recommendation on a consensus opinion of experts [7].

### Comorbidities in INPH

#### Musculoskeletal conditions

Musculoskeletal problems are a common comorbidity in INPH patients. The extent to which musculoskeletal conditions contribute to disabilities in gait and balance should be assessed routinely when examining patients with suspected INPH. When significant co-existing musculoskeletal conditions are confirmed, management needs to be individualised to the patient’s condition. Note that prognostic tests such as high volume tap test, external lumbar drainage and infusion tests sometimes yield false results if the CSF flow is altered because of a severe spinal canal stenosis [8]. Management may differ considerably depending on whether symptoms are primarily attributable to musculoskeletal comorbidities versus INPH. In such cases, a multidisciplinary approach may be required to optimise the management plan.

##### Conditions of the hip or knee

Most patients with INPH are elderly when diagnosed and may have a degree of osteoarthritis of the hip and/or knee. Other conditions such as gluteal enthesopathy, trochanteric bursitis or gait abnormalities caused by inadequate joint mobility are also common. The hallmark of all of these conditions is pain. While they may limit tandem gait, or cause unsteadiness and falls, they produce pain particularly with weight bearing. Provocative testing may also elicit pain but the diagnosis is usually confirmed through investigations such as x-ray examination, SPECT bone scan and more commonly MRI of the hip or knee.

Patients with INPH and painful hip or knee pathology should have treatment tailored to their clinical picture. The severity of pain will need to be balanced against the severity of the symptoms attributable to INPH, as well as the likelihood of response to shunting and candidacy for long-term rehabilitation. For example, patients with severe cognitive disturbance due to INPH may be unable to participate in a rehabilitation program necessary for successful outcome of hip or knee replacement surgery. In cases where prognostic tests for shunt responsiveness are positive and pain is not severe, treatment of INPH should take priority. However, if hip or knee pain is severe and cognitive impairment is mild, the hip or knee should be given priority for management.

Hip and knee prostheses are commonplace in the elderly. The presence of such prostheses is not usually a contra-indication to investigation or treatment of INPH. Patients with prostheses who have hip or knee pain, particularly pain with weight bearing that interferes with gait, should have their prostheses investigated to exclude complications such as loosening or peri-prosthetic fractures.

Hip fractures, especially in women, are common in the elderly. Recuperation time to maximal recovery after a hip fracture regarding gait and balance is about 6–9 months [9]. Gait and balance are important for the diagnosis of INPH and to evaluate outcome of surgery. It may therefore be reasonable to postpone shunt surgery for INPH for six months after a hip fracture if the patient is going to be included in prospective research projects.

##### Cervical spondylotic myelopathy

Cervical spondylotic myelopathy is caused by degenerative changes in the cervical spinal canal. In the elderly, this is the most common cause of impaired function of the spinal cord. Like INPH, it is typically painless and has an insidious onset. Early symptoms tend to be weakness, sensory changes and gait disturbance. Along with spastic gait, balance is often adversely affected by cervical myelopathy.

Cervical myelopathy may progress rapidly, often affecting upper limb function significantly as well as gait, and represents a significant risk in some patients undergoing surgery for other reasons. Endotracheal intubation with neck extension, positioning with the head rotated for shunt surgery and relative hypotension that often accompanies general anaesthesia, all heighten the risk of shunt surgery and may cause clinical deterioration in cases of existing cervical canal stenosis and myelopathy [10]. Accordingly, when a diagnosis of myelopathy is confirmed through MRI, priority is usually given to its treatment, particularly when there is associated myelomalacia.

##### Lumbar canal stenosis

Lumbar canal stenosis causes neurogenic claudication. This is a limitation of walking (or standing) for prolonged periods due to lower limb pain. Many patients do not have pain but rather lower limb paraesthesia or numbness. Sitting, usually for a short period, provides relief from symptoms and the patient can walk or stand again for a period of time. Except in severe cases of lumbar canal stenosis, walking is not affected in the initial stages as opposed to INPH where initiation of gait is a predominant symptom. Management of probable INPH should take priority over lumbar canal stenosis. The decision should be based on clinical symptoms rather than spinal radiological findings, which tend to be a poor predictor of clinical symptoms [11]. The reason for giving management of INPH priority is that lumbar canal stenosis will usually not limit gait unless the patient is able to walk significant distances. An exception to this scenario is the patient with lower limb symptoms due to lumbar canal stenosis at rest that can occur in severe cases and may indicate the rare but possible progression to paraplegia.

##### Other pathologies including stroke

There are several other musculoskeletal pathologies that should be considered in the assessment of patients with INPH. A hemiplegic gait often indicates previous stroke. Peripheral neuropathy, myopathy and polymyalgia rheumatica are other pathologies that need to be considered in the diagnosis. These are usually more easily assessed in cases where cognitive impairment is mild and where the patient is able to mobilise for some distance. Most difficulties arise where the patient is bed-bound or has difficulty describing other clinical symptoms, particularly pain. Diagnosis must be guided by a history from family members and careful clinical examination supplemented by other investigations.

In a recently-developed hemiplegia in which there is a clear cause it would be unusual to shunt a patient for INPH. However, other patients with more long-standing deficits and newly-developed hydrocephalic symptoms may benefit from shunting. Patients with deficits due to polio would be another example, although in these cases post-polio syndrome needs to be considered. In each of these cases, treatment should be decided based on the merits of the individual case.

##### Recommendations

1. If signs and symptoms of myelopathy are found in a patient being investigated for INPH, a spinal MRI should be performed (*Class I*).

2. If symptomatic and progressive, treatment of cervical spondylotic myelopathy should usually take priority over testing for INPH or shunt surgery (*Class IIa*).

3. Patients who have coexisting INPH and lumbar canal stenosis should usually undergo shunt surgery. Exceptions are patients with pronounced neurological symptoms due to stenosis, such as paraplegia who should undergo back surgery first (*Class IIb*).

4. A hip or knee prostheses is not a contra-indication to investigation or treatment of INPH. Patients with INPH and painful hip or knee pathology should have treatment tailored to their clinical picture.

5. In the research setting, INPH patients with hip-fracture may need to recover for 6–9 months before enrolling in INPH clinical studies in order to obtain valid outcome assessments (*Class IIb*).

#### Urinary problems

Urinary disturbances are common and distressing problems in the elderly. In adults, the prevalence of urinary tract symptoms has been reported to be 17% and it increases with age [12]. As urgency, frequency and incontinence are cardinal features of INPH, it is important to determine if the patient’s urinary symptoms are related to hydrocephalus. However, urgency with or without incontinence is not specific for INPH. The possibility of co-existing types of incontinence or urinary problems caused by other neurologic or non-neurologic disorders should be taken into consideration.

The investigation of urinary problems in INPH should include a detailed history targeting the most relevant signs and symptoms. Patients should be asked about urgency, frequency, nocturia, incontinence and any accompanying neurological symptoms. When symptoms are present, urinary tract infection and a large post void residual urine should be excluded. Patients can be asked to fill out a bladder diary for several days to provide information about how often they urinate, the volume of urine produced, and any episodes of incontinence. Voiding more than 8–12 times in a 24 hour period is usually considered compatible with a diagnosis of overactive bladder (below), but the frequency is also correlated to intake of fluids [13].

##### Frequency and urgency

There are five major types of urinary incontinence; (1) urge incontinence; (2) stress incontinence; (3) mixed incontinence; (4) overflow incontinence and (5) incontinence caused by other factors such as decreased mobility, cognitive impairment or medications.

Urge incontinence is caused by a sudden, involuntary bladder contraction and is also called “detrusor overactivity” or simple “overactive bladder”. This condition is defined as urinary urgency, often mixed with frequency or nocturia, with or without incontinence [13]. It could be caused by many of the most important diseases that co-exists with INPH: different types of dementia, stroke, small vessel disease and leukoaraiosis, Parkinson’s disease, multiple system atrophy and myelopathy [14]. Also common non-neurologic disorders such as prostatic hypertrophy, tumor and cystitis could give similar symptoms.

Stress incontinence is when the bladder and urinary sphincter cannot handle the increased compression during exercise, coughing, or sneezing, resulting in loss of urine. This kind of incontinence happens mostly to women with pelvic-organ prolapse [15] but also in men after prostate surgery.

Mixed incontinence is a combination of both stress and urge incontinence, and is most common in older women. Overflow incontinence, in which the bladder becomes distended and cannot be fully emptied, is rarer and is the result of bladder obstruction or injury. In men, it can be the result of an enlarged prostate.

Increased age, impaired functional status, increased duration of disease, diabetes and cholinesterase inhibitors are associated with an increased frequency of urinary incontinence [16]. Functional incontinence is a condition related to dementia and/or gait disturbance interfering with independent toileting skills.

##### INPH and urinary incontinence

The frequency of urinary problems in INPH is reported to be between 55% to 79% [3,5,6,17]. Storage symptoms (93%) are more common than voiding symptoms (71%) [18]. In a urodynamic study, it was reported that bladder capacity was small and detrusor overactivity seen in 95% of INPH patients; thus, detrusor overactivity is postulated to be the basis for most urinary urgency/frequency and incontinence in INPH. Improvement of bladder hyperactivity after lumbar tap test in INPH patients has been reported [19].

Recent perfusion studies with single photon emission tomography revealed that a hypoperfusion was present in the right frontal area in INPH patients with moderate or severe incontinence, compared to those with none or mild incontinence [20].

##### Recommendations

1. Detrusor over activity with urgency is a common urinary problem in INPH.

2. A carefully-obtained history of symptoms is important to the evaluation of urinary problems in INPH. If possible, a bladder diary should be obtained from the patient. Investigations should include tests for urinary tract infection and increased post-void residual urine (*Class I*).

3. In contrast to the gait disturbance and dementia, studies of urinary incontinence in INPH are scarce. Further studies are needed.

#### Vascular disease

An association between INPH and cerebrovascular disease is well supported. Neuropathological studies of the brain of hydrocephalic patients show signs of cerebrovascular disease such as sclerosis of the arterioli [21-23]. Hypertension and sometimes other vascular risk factors and a previous history of cerebrovascular incidents are often found in INPH patients [24-27]. Leukoaraiosis of the periventricular and deep white matter is common [28,29]. The cognitive deficits seen in NPH frequently involve accentuated impairment of frontal systems, a pattern shared with subcortical vascular cognitive impairment [24].

##### Risk factors

A high burden of vascular risk factors means that an individual will have an increased risk of developing manifest end organ damage in the form of stroke, white matter lesions, vascular dementia, ischemic heart disease or claudication. All these are common in the elderly and occur frequently as comorbidities in patients with INPH.

It is presently unclear whether vascular risk factors are an inherent part of the pathophysiology of INPH, accelerate the progression of the disease, or occur coincidently in INPH without affecting the hydrocephalus itself. It is also uncertain whether INPH symptoms improve if these risk factors are treated by lifestyle modifications or by medical interventions. Despite these uncertainties, it is important to assess and mitigate a patient’s likelihood of developing risk factor-related complications when INPH is diagnosed and whenever shunt surgery is considered.

A few small case–control studies regarding INPH and risk factors have been published [25,26,30]. The main finding is that hypertension is more common in INPH patients compared to matched controls with various other neurological or neurosurgical diseases. These studies are more than 20 years old and since then, the definition of hypertension has changed and the systolic blood pressure is today considered a much stronger risk factor than the diastolic blood pressure [31]. In the hydrocephalus field, the arterial pulse pressure and the arterial pulsations in the ICP curve have gained increasing interest. In healthy individuals, arterial pulse pressure appears to contribute to the expansion of the brain ventricles [32]. The amplitudes of cardiac-related ICP pulsations have been suggested as a promising predictor of good outcome after shunting patients with INPH [4,33].

Vascular risk factors other than hypertension may be associated with INPH but have yet to be confirmed. Several very large epidemiological studies have examined the effects of vascular risk factors on long-term prognosis in the elderly population. Among the most important of these trials are the “Interheart” and “Interstroke” studies that used case–control designs to investigate the association between known and emerging risk factors for ischemic heart disease and stroke [34]. In the “Interstroke” study involving 3000 patients and 3000 controls, 90% of stroke was predicted by a combination of 10 risk factors (history of hypertension, current smoking, waist-to-hip ratio, diet risk score, regular physical activity, diabetes mellitus, alcohol intake, psychosocial stress and depression, cardiac causes and ratio of apolipoproteins B to A1) [34]. It is reasonable to hypothesise that in a similar way, a risk factor profile is valid also in INPH, but additional research will be needed to confirm or reject this hypothesis.

Thus, there is a need to verify if those “new” vascular risk factors also implicate an increased risk for INPH as well as to better characterize its association with traditional risk factors such as hypertension. Future case–control and prospective cohort studies should not only test for possible associations between risk factors and INPH but also address the magnitude of risk factor abnormality that promotes the association. For example, it might be asked what degree of elevation in blood pressure or pulse pressure constitutes a risk in the context of INPH.

While a single risk factor can lead to a poor outcome, multiple vascular risk factors can act synergistically and worsen the prognosis to a greater extent than the simple additive effects of the individual risk factors. To capture this in future INPH studies, it would be valuable to calculate a total or global cardiovascular risk score for each patient. Various rating scales are available. One often used is the SCORE chart, which estimates the risk of dying a cardiovascular death within a decade [35] or the Framingham vascular risk scores [36].

##### Recommendations

1. Hypertension is the best-documented risk factor associated with INPH.

2. Modifiable vascular risk factors in INPH patients such as hypertension and hyperglycemia should be identified and treated prior to shunt surgery (*Class IIb*).

3. It is currently unknown if remediation of modifiable vascular risk factors will increase survival in INPH or improve hydrocephalus symptoms (with or without a shunt).

4. Traditional risk factors and cardiovascular disease should be identified in INPH patients, both in research and clinical practise. For the estimation of the global, or total, cardiovascular risk and its impact on prognosis, standardized and validated scales should be used (*Class IIb*).

5. Further studies are needed to better characterize the vascular risk factor profile of INPH. Established and emerging risk factors should be evaluated. It is important that future studies include large number of patients from different parts of the world with adequate (preferable population-based) controls.

##### Binswanger disease

Patients with vascular risk factors and extensive MRI white matter hyperintensity changes frequently present with symptoms and signs similar to those seen in INPH patients. These patients often fulfil the criteria for Binswanger Disease (BD) [37,38], a type of vascular dementia characterised by progressive and extensive subcortical ischemic vascular disease [38-40]. Ventricular enlargement is not a criterion of BD but is frequently reported in the later stages of the disorder [39]. Hence, late stage BD can be difficult or even impossible to distinguish from advanced stages of INPH.

Small vessel disease with periventricular white matter changes has been proposed to be the cause of INPH in a subgroup of patients with a less favourable prognosis [27,41,42]. Several studies, however, indicate no negative impact of vascular white matter changes on the effects of shunt surgery [42,43]. Moreover, it has been reported that patients fulfilling the criteria for BD with enlarged ventricles, extensive vascular white matter changes and symptoms compatible with INPH who had negative responses to infusion and drain prognostic tests, nevertheless showed improved motor and psychometric functions after shunt surgery [44]. In the aggregate, INPH and BD can co-exist and may even be associated. Therefore, it is important to consider shunt surgery also in patients showing evidence of co-existing cerebrovascular disease (CVD).

##### The prognostic implications of cerebrovascular disease in INPH

There is currently no predictive test that can reliably identify all INPH patients whose symptoms will be shunt responsive. Infusion studies showing an increased resistance to CSF outflow, or transient symptomatic improvement after CSF drainage predicts a good outcome after shunting [45,46]. However, a low resistance to outflow or a negative CSF drainage test may still be associated with improvement after shunting [47]. The value of these prognostic tests in the context of comorbidities is unknown.

Cerebrovascular disease can be associated with irreversible brain damage. In order to elucidate the prognostic importance of cerebrovascular disease comorbidity in INPH, consensus criteria for characterisation of study patients with co-existing CVD should be used, including measures that describe the amount of cerebrovascular pathology. Using MRI and volumetric segmentation of periventricular and deep white matter hyperintensities, structural changes associated with CVD can be measured in research study populations. These structural changes, however, have a limited predictive value and cannot be used to select candidates for shunt surgery [42-44,48]. The cerebrovascular disease process causes metabolic changes in the brain of varying patterns that are more pronounced than in normal ageing [49,50]. Metabolic impairment is also important in INPH, probably reflecting a brain dysfunction that may be reversed by shunting [51]. MR diffusion weighted imaging (DWI), perfusion weighted imaging (PWI) and diffusion tensor imaging (DTI) are all promising techniques that can be used to study functional and metabolic changes in the white matter in order to identify imaging patterns associated with favourable outcome after shunting.

The CSF is in connection with the white matter extracellular fluid. Therefore, analysis of CSF biochemical markers is appropriate to describe different pathophysiological changes in this brain region. CSF biomarkers associated with cerebrovascular disease show increased levels in some INPH patients, but their predictive value in relation to the outcome after shunting has yet to be established. High preoperative CSF levels of the axonal marker NFL, have been reported to correlate with a favourable outcome after surgery in INPH [52]. Reports on the diagnostic value of CSF sulfatide in distinguishing between INPH and BD are contradictory [53,54]. A combined pattern of NFL, P-tau, and Abeta42 has been reported to distinguish between the clinical diagnoses of INPH and BD [53], a finding that needs to be confirmed. Further studies are needed linking CSF biomarkers of vascular disease to outcome of shunt surgery.

Future research studies should focus on elucidating the importance of CVD comorbidity in INPH patients including the possible prognostic value of CVD. It is important that study inclusion criteria liberally allows inclusion of patients with evidence of CVD, regardless of severity, and that studies follow up outcome after shunting in a standardised way. In order to find better predictive tests, patients with different degrees of CVD burden and different degrees of shunt outcome must be compared.

##### Recommendations

1. Among cerebrovascular disorders, BD is the most important for INPH differential diagnosis, since these disorders often show similar clinical and radiological signs.

2. Subcortical CVD frequently co-exists with INPH. CVD patients often have vascular MR white matter changes and may also present with focal neurological signs or vascular cognitive impairment.

3. INPH patients with evidence of CVD may improve significantly after shunting (*Class IIa*).

4. Better predictive tests that can select shunt candidates in patients with mixed INPH and BD are needed.

5. A consensus classification of coexisting INPH and CVD is needed where both clinical and neuroimaging measures such as MRI are included.

6. Future clinical studies should liberally include INPH patients with co-existing cerebrovascular disease and evaluate the shunt effect in this subgroup.

7. Future research should focus on assessing pathophysiological changes characteristic of INPH and CVD using MR diffusion and perfusion imaging as well as CSF biomarkers related to clinical measures at baseline and changes after shunt surgery.

#### INPH and alzheimer’s disease

Alzheimer’s disease (AD) is the most common cause of dementia and a common comorbidity with INPH. Recently, guidelines defining AD were published emphasizing history, neuropsychological testing and clinical examination as the main base for diagnosis [55]. To increase the diagnostic accuracy, typical AD histological features of β-amyloid plaques and neurofibrillary tangles can be confirmed either directly by a brain biopsy [56] or indirectly by biomarkers such as the phosphorylated tau/amyloid beta 1–42 ratio in CSF [57]. Positron emission tomography (PET) amyloid imaging and MR volumetry are also promising methods to support AD diagnosis, particularly for research studies.

Although AD itself is not usually associated with a gait disturbance at presentation, gait disturbances are common in the elderly population that is most prone to develop AD. This fact, combined with the occurrence of cognitive impairment and ventriculomegaly in both AD and INPH, can make differential diagnosis of these two conditions challenging.

INPH and AD can occur together in a mixed form, INPH-AD [58]. Although these disorders have overlapping symptoms, a careful history and examination by a skilled physician or neuropsychologist can assist in their differential diagnosis as comorbid illnesses. Rapid forgetting of newly acquired information related to hippocampal dysfunction is the most common presenting symptom of Alzheimer’s disease. Although memory may also be affected in INPH, it is usually as a manifestation of frontal executive disturbances that can often be improved by cues or reminders. Various neuropsychological tests to differentiate AD and INPH have been suggested [59,60]. Progressive disturbances of gait and balance are common presenting symptoms of INPH, whereas comparable degrees of motor impairment tend to occur only late in the course of AD [61]. Likewise, urinary frequency, urgency and incontinence can occur in early stages of INPH but tend to be late stage symptoms in AD.

The similarity between cognitive impairment in vascular dementia and that seen in INPH patients is another potential diagnostic confounder. Moreover, also AD and vascular dementia can co-exist in a single patient. Further research is needed to develop techniques to better differentiate INPH from mixed INPH-AD. Future clinical trials should endeavor to identify the frequency of mixed INPH-AD in the study cohorts.

##### Brain biopsy

AD-related neuropathological changes have been found in biopsies from patients with “amnestic mild cognitive impairment” (MCI) [62], i.e., a prodrome to AD characterized by subtle memory problems occurring without a significant decline in activities of daily living. It is believed that the AD pathology in those patients develops many years before onset of the manifest AD dementia [63]. However, “typical” AD features have also been found at autopsy in elderly patients who did not develop cognitive decline before their death [64,65].

In INPH patients, it is possible to obtain a small cortical brain biopsy at the time of shunt placement or in the course of carrying out invasive pre-operative recording of intracranial pressure [66]. Biopsies obtained in this manner show typical AD histological findings in 25-40% of INPH patients [67-70]. It is unclear whether these findings correspond to manifest AD, a pre-stage of AD or if they are random findings.

Studies have also attempted to correlate the degree of AD findings at biopsy with outcome after shunt surgery. Patients with AD findings at biopsy can still improve after shunting. The available information indicates that the presence of AD pathology does not affect survival after shunt placement, but is associated with a lesser degree of improvement compared to INPH patients lacking AD pathology [61,67,68,70-72]. More studies are needed to clarify and confirm these relationships.

In summary, brain biopsies open a novel window to the study of the pathobiology of INPH and its relation to other neurodegenerative diseases like AD. Brain biopsy is associated with a small risk of adverse events such as infection, bleeding or epilepsy. As such, it should only be performed in research protocols approved by an ethical review board.

#### CSF biomarkers

In AD, there is an accumulation of amyloid β (Aβ) in the brain that can be reflected in alterations of the amyloid content of ventricular CSF. CSF from INPH patients is accessible via lumbar puncture or through a puncture of the lateral ventricle. CSF biomarkers that can be used in support of an AD diagnosis include total tau protein (T-tau), hyperphosphorylated tau protein (P-tau) and beta-amyloid protein (1–42) (Aβ42). A typical AD pattern consists of a decreased Aβ42 and an increased T- and P-tau [73] in the CSF. Limitations are the lack of uniform reference values and the fact that T- tau is increased in several brain disorders [74]. Recently, standards for biomarker collection for AD studies were published that may also be applicable to future studies of CSF biomarkers in INPH [57].

In two studies comparing CSF biomarkers in INPH versus healthy controls, Aβ42 concentrations were reduced but T- and P-tau were normal in INPH subjects [75]. In direct comparison between INPH and AD, Aβ42 was reduced in INPH but had varying results regarding tau protein [76-78]. Increased [79] or normal [76,78] tau levels were found in INPH. A positive correlation between tau protein and the severity of dementia in INPH has been reported [79]. It has not been possible to use CSF biomarkers to predict response to shunt surgery [80]. A major problem with these studies is that the number of clinically diagnosed INPH-AD is unknown or not mentioned, making comparison between studies difficult.

In a large study consisting of 182 patients with presumed NPH, findings at brain biopsy correlated with the concentration of Aβ42 and tau in the CSF [81]. However, more studies are needed before it is possible to make general recommendation on the use of CSF biomarkers in the clinic to identify mixed cases of INPH-AD [82]. The distinctive alterations in ventricular size [83,84] and CSF dynamics associated with INPH have been shown to alter the concentration gradients of monoamine metabolites in CSF of hydrocephalus patients [85,86] and could alter the concentrations of other CSF biomarkers in unpredictable ways.

##### Recommendations

1. INPH and AD can co-exist. The frequency of clinical manifest INPH-AD is unknown, but it may be common. Patients with suspected INPH and cognitive impairment should therefore be routinely evaluated for possible AD by a clinician skilled in the diagnosis of neurodegenerative disorders (*Class IIa*).

2. Patients presenting with an isolated dementia syndrome rarely have INPH. Those patients with a gait disturbance in the absence of cognitive decline rarely have AD. Better techniques are needed to identify INPH-AD patients and to determine the degree to which neurodegenerative changes versus hydrocephalus-related pathology contributes to disability in individual cases.

3. CSF and neuroradiological biomarkers for AD have excellent potential as supplemental diagnostic, prognostic and therapeutic indicators in the assessment of INPH patients. However, further studies are needed to better define sensitive and specific imaging parameters and biomarker alterations that are characteristic of INPH.

4. Small cortical brain biopsies are a promising investigation tool in INPH research, particularly for identifying mixed INPH-AD. The clinical value of biopsies has to be validated and for now brain biopsies should only be performed in the context of research studies (*Class IIb*).

5. Patients with INPH-AD can improve following shunt surgery. However, outcomes (particularly cognitive) may be less satisfactory than in “typical” INPH patients.

#### Other dementias

Gait disturbances are a cardinal feature of INPH and several neurodegenerative disorders. Disorders that can pose challenges to the differential diagnosis of INPH include Parkinson’s disease (PD) and Parkinsonian syndromes including dementia with Lewy bodies (DLB), corticobasal syndrome (CBS), progressive supranuclear palsy (PSP), multiple system atrophy (MSA) and a subset of patients with frontotemporal dementia (FTD). These neurodegenerative diseases tend to increase in incidence with age, as does INPH. The size of the cerebral ventricles also increase with age. Because of these overlapping features it is not uncommon for persons with idiopathic PD or secondary Parkinsonism to be suspected of having hydrocephalus. This section will address each diseases and ways of diagnosing the neurodegenerative comorbidities in patients with suspected INPH.

##### Parkinson’s disease

The major triad of PD is tremor, rigidity and akinesia. The best features for differentiating PD from other Parkinsonian diseases is a careful history and clinical evaluation revealing asymmetry of symptoms and signs, resting tremor and response to levodopa [87]. Specifically, the evaluation should look for Parkinsonian features affecting the face and arms more than or as much as the legs, or are asymmetrical, or accompanied by tremor or vertical eye movement abnormality, or in a patient with disinhibited behavior. In INPH, lower body Parkinsonism is characteristic.

##### Dementia with lewy bodies

The published diagnostic guidelines for DLB [88,89] include progressive dementia, Parkinsonism, hallucinations, fluctuations in attention and arousal, rapid eye movement (REM) sleep disorder, neuroleptic sensitivity and low dopamine transporter activity on SPECT or PET scan. While INPH patients have gait difficulty and dementia, Parkinsonism affecting upper body, visual hallucinations and REM sleep disorder will help distinguish DLB from INPH.

##### Corticobasal syndrome

Features of CBS include asymmetrical apraxia, alien limb, cortical sensory loss, asymmetric myoclonus, apraxia of speech and non-fluent aphasia [90]. Because patients with this syndrome may have striatal involvement the ventricles may appear large and the patients may have Parkinsonism but the asymmetry of the cortical symptoms usually distinguishes this syndrome from INPH.

##### Progressive supranuclear palsy

Patients with PSP [91] have voluntary vertical gaze impairment, axial rigidity, early falls, difficulty swallowing, speech impairment. In the early stages movement disorder specialists may have difficulty distinguishing PSP from PD but over time the diagnosis becomes more obvious. It is important to evaluate eye movements in possible INPH patients. Further, speech and swallowing difficulty are not features of the early stages of INPH.

##### Multiple system atrophy

MSA can present with a cerebellar or a Parkinsonian form [92]. The latter is more common. Autonomic dysfunction such a postural intolerance, bladder or bowel dysfunction, impotence and sweating dysfunction are frequently present. As many as half the patients may suffer REM sleep disorder. The subset with cerebellar degeneration has ataxia. Other features include inspiratory stridor and dysarthria. The cerebellar dysfunction and autonomic features and speech changes should help distinguish INPH from this disease.

##### Frontotemporal dementia

Patients with FTD are classified into behavioral variant (bv) [93], non-fluent progressive aphasia (nfpa) and semantic dementia (sd) subtypes [94]. The bvFTD patients have features of behavioral disinhibition, apathy, loss of empathy, perseveration, hyperorality and a neuropsychology profile showing executive dysfunction with relatively preserved memory and visuospatial dysfunction. The nfpaFTD patients have progressive impairment in word finding and other aspects of expressive language. sdFTD patients lose the meaning of words and other aspects of semantic knowledge. Because FTD patients frequently have Parkinsonism and may have caudate atrophy, that makes the frontal horns of the ventricles appear enlarged, they may be thought to have a component of INPH. However, language and behavioral disturbances in INPH are generally milder and less evidently progressive than in FTD, and these differences can be useful in distinguishing among these conditions.

#### Psychiatric and behavioral disorders

Cognitive symptoms and neuropsychological dysfunction are common in patients with INPH. Disturbances in executive and memory function usually dominate and impaired wakefulness may be present, but a range of other less typical symptoms have been reported, such as anxiety, emotional instability and motivational blunting, depression, impatience and psychosis [24,41,95-100], some of which have been shown to improve after shunting. However, little is known about the frequency of psychiatric symptoms in INPH or the frequency of co-existing psychiatric or behavioral disorder. Using neuropsychological testing, INPH patients can be distinguished from healthy individuals [99]. No such testing is available for differentiating between INPH and psychiatric or behavioral disorders and there are no biomarkers available. As a consequence, it may be difficult to differentiate between psychiatric features of INPH and symptoms caused by other psychiatric disorders. A premorbid history of psychiatric illness such as depression or psychosis may be helpful.

In the work-up of patients, it is essential to search for and evaluate the typical cognitive symptoms of the INPH disorder using neuropsychological testing as well as assessing the typical motor and urinary symptoms. If a psychiatric or behavioral disorder is suspected, the patient should usually be referred to a psychiatrist. A psychiatric disorder should primarily be suspected in patients presenting with psychosis. Depression with mental slowness, apathy or anxiety may mimic hydrocephalic symptoms and underlying depression is important to rule out or treat. Patients with suspicion of INPH presenting with major depressive symptoms should therefore be carefully evaluated using an appropriate evaluation instrument and given a trial of antidepressant medication for a period of time before the final decision to perform shunt surgery is taken. This approach is taken to rule out depressive symptoms mimicking INPH and to allow proper assessment of cognitive symptoms and improvement after shunting. At the same time, depression, anxiety, psychosis and other behavioral symptoms may be masked by apathy and cognitive deficits caused by INPH and such symptoms may therefore be aggravated after successful shunting. Doses of anti-depressant and other psychoactive medications should be monitored closely in INPH patients and adjusted appropriately after shunt surgery.

##### Recommendations

1. Very little is known about frequency of depression and other psychiatric and behavioural disorders in INPH but a wide range of symptoms have been described in these patients. Population based case–control and cohort studies are needed as well as studies of biomarkers and neuropsychological testing.

2. In the clinical work-up, effort should be put on identifying and measuring cognitive and neuropsychological dysfunction characteristic of INPH (*Class I*).

3. A comorbidity of psychiatric or behavioural disorder should not exclude a patient from shunt surgery but objective and balanced information of expected effect of shunting is essential (*Class IIa*). Depression, anxiety or psychosis in INPH are optimally treated before surgery (*Class I*).

4. A primary depressive disorder should be ruled out in suspected INPH patients presenting with major depression symptoms. Severity of depression should usually be assessed using a depression rating scale and patients should be treated with antidepressant drugs before a probable diagnosis of INPH can be made (*Class IIa*).

##### Brain imaging

Structural brain imaging by computed tomography (CT) and magnetic resonance imaging (MRI) is essential for screening and clinical diagnosis of INPH. CT is convenient, fast and inexpensive, and very well suited to reveal ventriculomegaly. However, MRI has become the first choice in the preoperative investigation of INPH as it is essential for the differential diagnosis of disorders, such as BD or AD. While an enlarged ventricular system is a prerequisite, certain imaging signs favour INPH, however none have proved sensitive enough, including flattening of the cortical sulci, widened temporal horns, an enlarged third ventricle, callosal angle ≤ 90 degrees and an increased aqueductal flow.

Although ventricular dilatation is a hallmark of hydrocephalus, it is also a sign of cortical atrophy. A primary aim is to differentiate between ventricular dilatation caused by a CSF dynamic disturbance and dilatation caused by a primary cerebral atrophy, which can be difficult, especially in mixed cases. Coronal sections are particularly useful in evaluating the high cerebral convexity and medial temporal lobe including the hippocampus. It has been observed that in patients with INPH, the subarachnoid space in the Sylvian fissures is dilated (or at least not narrowed) while at the same time those over the high cerebral convexity and medial surface are narrowed [101]. This disproportionately enlarged subarachnoid space hydrocephalus (DESH) [101] has been reported to differentiate INPH from brain atrophy in Alzheimer’s disease, results that need to be confirmed. In addition, hippocampal atrophy [102] and widening of the parahippocampal sulci [101] are usually milder in INPH compared with AD. Importantly, there are INPH patients displaying a few large convexity sulci (“focally dilated”, “entrapped” or “transport” sulci) [103,104] which should not be mistaken for cortical atrophy. Moreover, population-based studies indicate that some of the elderly present with MRI features consistent with DESH without any neurological symptoms [105].

In frontotemporal dementia (FTD), the frontal cortical atrophy is typically marked, which together with reduced frontotemporal cerebral blood flow and metabolism makes it possible to differentiate FTD from INPH. Periventricular and deep white matter changes (leukoariosis) are seen more often and more severely in patients with INPH than in healthy elderly individuals; however, they are not requisite signs for INPH and rather suggest comorbid chronic cerebral ischemia [106].

Periventricular and deep white matter changes are discussed in section 2.3.4. New imaging techniques represent a promising development, such as diffusion or perfusion weighted MR imaging, MR spectroscopy or cerebral blood flow or metabolism measurement. These techniques may be used to characterise pathophysiologically different types of white matter changes but have so far not proven able to reliably identify reversible changes typical for INPH or to predict outcome after shunting [107-109]. More studies using contemporary techniques are needed where inclusion of patients with vascular changes must be liberal.

Cerebral microbleeds are probably a marker of cerebral microangiopathy [110]. The microbleeds are best visualized on T2* gradient echo or susceptibility-weighted MR imaging and are reported in patients with vascular dementia but also in healthy elderly. Cerebral microbleeds may play a role in the pathophysiology of INPH and CVD but have so far not been studied in patients with INPH.

##### Recommendations

1. MRI of the brain is essential for the evaluation of comorbidity in INPH (*Class I*). For patients who cannot have an MRI, CT scanning with axial, sagittal and coronal views is recommended.

2. Coronal MRI is useful for the evaluation of the morphological changes of the brain characteristic of INPH and other degenerative dementias. The sign of tight high-convexity and midline subarachnoid spaces (i.e., “DESH”) can be helpful in differentiating INPH from brain atrophy in AD. Hippocampal atrophy and widening of the parahippocampal sulci are suggestive of comorbid AD.

3. The role of white matter hyperintensities in INPH is unclear. Patients with extensive white matter lesions may improve following surgery, but the relation between its severity and the magnitude of shunt effect is unclear.

4. Cerebral microbleeds have not been studied in INPH.

### How to investigate and quantify comorbidity

The overall type and degree of comorbidity in INPH should always be assessed. A comorbidity index created only for INPH would probably have advantages. Such a scale consisting of vascular risk factors, peripheral vascular disease, cerebrovascular disease, heart disease etc. has been suggested [111]. However, it has not been validated and is not yet established. While waiting for a dedicated INPH index, a universal scale could be helpful in clinic and research. Two of the most promising comorbidity scales that have been validated in the elderly are the Modified Cumulative illness rating scale (CIRS-G) [112] and the Charlson comorbidity index (CMI) [113-118]. These scales have not been created specifically for INPH, meaning that diseases are weighed and scored irrespective of the impact the individual disease has on the prognosis of INPH. However, they probably represent the best index to be used until a dedicated INPH scale has been accepted by the hydrocephalus community.

A cornerstone in the work-up of INPH patients is a detailed history and a thorough physical examination of clinical symptoms. By performing a careful examination, clinical symptoms typical for important potential co-morbidities will usually be discovered, such as Parkinson’s disease and related disorders, Alzheimer’s or other dementia types, or other neurologic or musculoskeletal disorders. Several auxiliary investigation methods are at hand for differential diagnostics and for quantification of co-morbidity in the single patient, the most important being displayed in Table 1.

**Table 1:** Investigations that may be performed to verify comorbidity in INPH

**Investigation**	**Indication**
MRI of the spine	Lumbar or cervical spinal stenosis?
MRI brain (T1, T2, FLAIR, T2*, diffusion, perfusion)	Atrophy? White matter lesions? Infarctions? Microbleeds? Brain volumes? Cerebral blood flow?
X-ray or MRI of hip or knee	Arthrosis?
Bladder diary	Type of urinary problems?
Measurement of residual urine	Obstruction?
Urinalysis/urine culture	Urinary tract infection?
Vascular risk factor analysis/inventory	Burden of cerebrovascular disease risk factors?
CSF T-Tau, P-Tau and Aβ42	Alzheimer’s disease?
Brain biopsy	Alzheimer’s disease. Only in research and if approved by internal review board
Depression scale	Treatment of depression?
Response to levodopa	Parkinson disease?
Dopamine transporter imaging	Dementia with Lewy bodies?
Test of autonomic dysfunction	Multiple system atrophy?
Comorbidity index/rating scale	Quantification of co-morbidity

### Conclusion

Comorbidity is an important factor in the prognosis and post-operative outcome of shunt surgery for INPH. Differences between INPH cohorts sampled at different hydrocephalus centers may be partly explained by variations in comorbidity across those centers. Identification of comorbidities should thus be a central part of the clinical management of INPH where a detailed history, physical examination, and targeted investigations are the basis for diagnosis and grading. Future INPH research should focus on the contribution of comorbidity to overall morbidity, mortality and long-term outcome. Investigators should also consider broader inclusion criteria in order to include rather than exclude patients with comorbidities so results can be more reliably extrapolated to the general population. We hope that this review will inspire investigators to create new hypotheses that will improve our understanding of comorbidity in INPH, to advance the field and for the benefit of INPH patients.

## Competing interests

JM has a patent interest in Likvor AB but no ownership, board function or funding. NG-R is on the Scientific Advisory Board and is consultant for Codman. MI, BTK, VL, EM, BO, MT and NR have no competing interests. MW is the associate editor for Ethics for Continuum, and president of the International Society for Hydrocephalus and CSF Disorders 2012–2014. He holds the following patents relating to shunt surgery: United States Patent 6,585,677, Canadian Patent 2,356,032, United States Patent 6,932,787 B2, Continuation of US Patent 6,585,677, International Patent WO 2006/060181 A1. He receives research support relating to hydrocephalus and shunt obstruction from NeuroDx Development, Trevose, PA (NCT01323764), from National Space Biomedical Research Institute, Project Numbers SMST02802 and CA02801 and has 5% interest in Mensana Therapeutics.

## Authors’ contributions

Authors contributed equally in the process of creating recommendations and the manuscript. All authors read and approved the final manuscript.
